# Observational Study Evaluating Blood‐Based Biomarker Use for Confirmatory Alzheimer's Disease Diagnosis in Real‐World Clinical Practice Within the United States

**DOI:** 10.1002/alz70856_099857

**Published:** 2025-12-25

**Authors:** Marwan N. Sabbagh, Daryl Jones, Jo Vandercappellen, Leah Zullig, Michelle M Mielke, Douglas R. Galasko, Nicholas Giordano, Christine Divers, Feride H Frech, Soeren Mattke, Harald Hampel, Hideki Toyosaki

**Affiliations:** ^1^ Barrow Neurological Institute, Phoenix, AZ, USA; ^2^ Eisai Inc., Nutley, NJ, USA; ^3^ Duke University School of Medicine, Durham, NC, USA; ^4^ Division of Public Health Sciences, Wake Forest University, School of Medicine, Winston‐Salem, NC, USA; ^5^ Shiley‐Marcos Alzheimer's Disease Research Center, University of California, San Diego, CA, USA; ^6^ PINC AI™ Applied Sciences, Premier, Inc, Charlotte, NC, USA; ^7^ University of Southern California, Los Angeles, CA, USA

## Abstract

**Background:**

The global rate of undetected dementia is high, and recently approved anti‐amyloid therapies for early Alzheimer's disease (AD) necessitate a timely and accurate diagnosis. High‐accuracy blood‐based biomarkers (BBMs), meeting the CEOi performance criteria,^1^ may provide accessible and scalable confirmation of amyloid pathology. This study will analyze the time to final biomarker‐diagnosis using confirmatory BBMs and current standard of care (SOC) biomarkers.

**Method:**

During this prospective observational study, we will collect secondary data (via electronic medical records) as patients progress through the AD diagnostic pathway at three sites within the United States. This study aims to evaluate the reach (ie, proportion and representativeness) of patients who receive a BBM‐confirmed diagnosis over a study period of 6 months, compared with the current SOC (cerebrospinal fluid or positron emission tomography); determine the time to biomarker‐confirmed diagnosis (over a study period of 6 months); and assess the number of visits required for diagnosis by type of test or procedure. The protocol has been approved by a central institutional review board.

**Result:**

Three memory care clinics have been recruited. Up to 180 patients aged ≥55 years, who present to one of three sites for further workup, with a clear history of progressive cognitive decline indicating a possible or suspected diagnosis of AD, will be recruited.

This descriptive analysis will summarize data collected using the electronic case report form, including cognitive assessments, laboratory tests/imaging, patient characteristics, confirmatory diagnostic test used, number of visits and time until diagnosis, other diagnostic procedure(s), and adverse events associated with any diagnostic test. The study is intended to be descriptive in nature.

**Conclusion:**

This study will describe time to final biomarker‐confirmed diagnosis for BBMs and other current SOC biomarkers. These findings will characterize the evolving patient journey through the AD diagnostic pathway, from diagnoses with BBMs and current SOC, possibly highlighting potential benefits of BBMs.

**Reference**

1. Schindler SE, et al. Nat Rev Neurol. 2024;20(7):426–439.

